# Enoxaparin dose reduction for thrombocytopenia in patients with cancer: a quality assessment study

**DOI:** 10.1007/s11239-017-1478-0

**Published:** 2017-02-16

**Authors:** Simon Mantha, Yimei Miao, Jonathan Wills, Rekha Parameswaran, Gerald A. Soff

**Affiliations:** Memorial Sloan Kettering Cancer Center, 1275 York Ave., Howard-717, New York, NY 10065 USA

**Keywords:** Thrombosis, Cancer, Anticoagulation, Thrombocytopenia

## Abstract

The development of thrombocytopenia in the setting of therapeutic anticoagulation for venous thromboembolic disease (VTE) is common in cancer patients, but guidelines for management are based on limited past data and have not been validated. In 2011, Memorial Sloan Kettering Cancer Center (MSKCC) implemented the following guidelines in this setting: administer full dose enoxaparin for a platelet count > 50,000/mcL, half-dose enoxaparin for a platelet count of 25,000–50,000/mcL, and hold anticoagulation for a platelet count < 25,000/mcL. We now report validation of safety and efficacy of these guidelines. As a Quality Assessment Initiative, we evaluated our guidelines for adult cancer patients at MSKCC who were on therapeutic-dose enoxaparin for VTE during the years 2011 through 2013 and experienced at least one 7-day period of thrombocytopenia (platelet count ≤ 50,000/mcL). We assessed adherence to the enoxaparin dose modification guidelines, major bleeding, clinically relevant non-major bleeding, recurrent VTE, and mortality during the thrombocytopenic episodes. We identified 99 patients with 140 episodes of thrombocytopenia of 7 or more days. The median duration of these thrombocytopenic episodes was 12 days. The enoxaparin dose was modified in 133 of the 140 episodes (95%), reflecting satisfactory adherence to our institutional guidelines. There were no recurrent VTE events or major bleeding episodes when the anticoagulant dose was reduced or held. In this cohort, there was only one major bleeding episode, a trauma-associated retroperitoneal hemorrhage that occurred on the third day of a thrombocytopenic episode, prior to enoxaparin dose modification. There were 13 clinically relevant non-major bleeding episodes. Lastly, 10 patients died of cancer-related causes during an episode of thrombocytopenia. This Quality Assessment Initiative supports the safety and efficacy of our guidelines for therapeutic enoxaparin dose modification.

## Introduction

Management of venous thromboembolism (VTE) is a common and challenging problem in cancer patients, with incidence rates ranging up to approximately 19% of patients, depending on the tumor type and therapy [1, 2]. Arterial and venous thrombosis remain a significant cause of cancer associated morbidity and mortality [3]. Low-molecular weight heparin (LMWH), such as enoxaparin or dalteparin, has been the recommended treatment of thrombosis in cancer [4, 5]. However, there are unique challenges to the safe and effective treatment of VTE in cancer patients. The rate of recurrent thrombosis and major bleeding is higher in the cancer population with VTE, compared with the general medical population [6]. In two studies of dalteparin for cancer-associated thrombosis, the 6-month rate of major bleeding was approximately 6–9% [7, 8].

One particular challenge in the management of thrombosis in cancer is the frequent occurrence of thrombocytopenia. Thrombocytopenia in the setting of cancer treatment is primarily secondary to chemotherapy, but also results from infection, liver metastases, and myelophthisis. Thrombocytopenia and anticoagulation are both independently associated with an increased risk of bleeding, but no prospective studies have been published to guide anticoagulation management during episodes of cancer-associated thrombocytopenia. The limited published experience has been relatively small case series [9, 10]. In 2009, Lee noted, “…there is little literature on the management of these difficult patients...” [11].

Based on the available literature at the time, in 2011 Memorial Sloan Kettering Cancer Center (MSKCC) implemented the following guidelines for the use of enoxaparin in the treatment of cancer-associated VTE: administer full dose for a platelet count > 50,000/mcL, half dose for a platelet count of 25,000/mcL to 50,000/mcL and hold drug temporarily for a platelet count < 25,000/mcL. As a Quality Assessment Initiative (QAI), we reviewed our institutional experience, to determine adherence to our guidelines, as well as the incidence of adverse outcomes, such as bleeding and recurrent thrombosis, and all-cause deaths.

## Materials and methods

### Patients and outcomes

We queried the clinical information systems for a list of all individuals treated at Memorial Sloan Kettering Cancer Center and meeting the following criteria: aged 18+ years, were prescribed therapeutic-dose enoxaparin, had at least one ICD9 code for VTE, and subsequently had at least one platelet count measurement below 50,000/mcL during the years 2011–2013. VTE and outcome events were confirmed by manual chart review. This analysis did not include patients after 2013, since in January of 2014 rivaroxaban was implemented as an alternative treatment option for patients with cancer-associated VTE at MSK. The subset of individuals with at least one 7 day period of documented thrombocytopenia (≤50,000/mcL) was selected for analysis. The electronic medical record was assessed to verify the doses of enoxaparin administered during the period of thrombocytopenia.

Standard full-dose enoxaparin is 1 mg/kg twice daily or 1.5 mg/kg once daily. Allowing for rounding of doses due to the use of pre-filled syringes, we considered doses of >1.125 mg/kg daily or >0.75 mg/kg twice a day as full dose. Clinical notes and radiology reports were reviewed to ascertain major and clinically relevant non-major bleeding (CRNMB) [12, 13], new or recurrent thrombosis, or death. This project was approved by the Quality Improvement Department at MSKCC and approved by the Institutional Review Board.

### Statistical analysis

Given the low numbers of events observed, we employed univariate analysis only. Considering that the assumption of normal distribution did not apply to thrombocytopenic episode-specific platelet count means, non-parametric statistical testing was done with the Kruskal–Wallis test. The R 3.2.3 for Windows software platform was used for all computations.

## Results

There were 15,022 individuals with an ICD9 code for VTE during the study period of January 1, 2011 through December 31, 2013. Of those, 4195 had been administered enoxaparin at least once, 4166 patients were at least 18 years of age as of the first day of the study period, 580 patients had at least one platelet count below 50,000/mcL and 367 of those subjects had been on full-dose enoxaparin at least once during the observation period. After review of the records of these 367 cases, 140 episodes in 99 patients were identified who sustained at least one episode of thrombocytopenia (≤50,000/mcL) lasting 7 days or more and were on full-dose enoxaparin before entering their platelet nadir. The cancer diagnoses of those patients are shown in Table 1.

**Table 1:** Distribution of cancer diagnoses

	Cases (N)	%
Cancer type		
Lymphoma	24	24.2
Leukemia	19	19.2
Multiple myeloma	11	11.1
Lung cancer	8	8.1
Sarcoma	7	7.1
Germ cell tumor	6	6.1
Other solid tumor	20	20.2
Other hematological neoplasm	4	4
Cancer stage for solid tumors		
0 (*carcinoma in situ*)	1	2.4
I	6	14.6
II	3	7.3
III	10	24.4
IV	13	31.7
NA	8	19.5

The median age for the cohort was 58.3 years and 66.7% were male. 58.5% were being treated for a hematological neoplasm, including lymphoma, leukemia and multiple myeloma. In 78.6% of thrombocytopenic episodes, chemotherapy had been administered within the prior month. Infection, myelophthisis, graft versus host disease or liver disease were less common causes of thrombocytopenia (see Table 2). Heparin-Induced Thrombocytopenia (HITT) was not found to be present in any of the cases. In review of medical records, in only seven patients was HITT suspected and heparin-dependent antiplatelet antibody levels tested. All 7 had negative values, (range 0.025–0.092, Normal <0.40 OD405).

**Table 2:** Distribution of causes for thrombocytopenia

Cause	N	%
Chemotherapy	110	78.6
Mixed causes	13	9.3
Infection	11	7.9
Myelophthisis	4	2.9
Graft versus host disease	1	0.7
Liver disease	1	0.7

In most cases, the index VTE episode leading to anticoagulation was a lower extremity DVT and/or a PE (see Table 3 for details). The median time from index VTE to episode of thrombocytopenia of 89.5 days. An inferior vena cava filter was present at the time of thrombocytopenia or inserted during the episode of low platelet count in 21 individuals.

**Table 3:** Characteristics of index VTE episodes

Characteristic	N	%
VTE event type		
Lower extremity DVT	37	37.4
Pulmonary embolism	26	26.3
Portal vein thrombosis	2	2
Upper extremity DVT	26	26.3
Other venous thrombotic episode	8	8.1
Median time to thrombocytopenia (days)	89.5	NA

*VTE* venous thromboembolic, *DVT* deep vein thrombosis

**Fig. 1:**
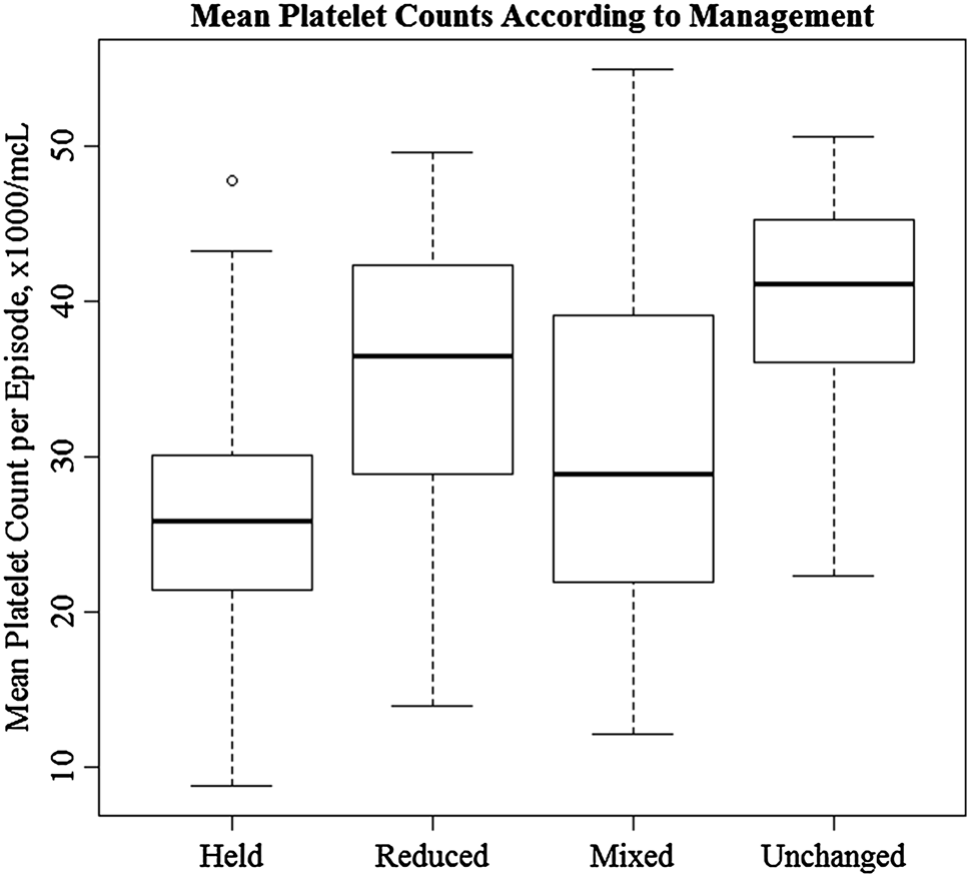
Mean thrombocytopenic episode-specific platelet counts according to management. The *top* of a box represents the 3rd quartile, while the *bottom* corresponds to the 1st quartile and the *line* within the box is the median. *Upper whiskers* correspond to the maximum value which does not exceed the 3rd quartile plus 1.5 times the interquartile range and *lower whiskers* are situated at the minimum value which is not lower than 1st quartile minus 1.5 times the interquartile range. *Circles* outside the whiskers represent individual outliers

The dose of enoxaparin was adjusted in 133 (95%) of thrombocytopenic episodes. The anticoagulant dosage was reduced in 20 episodes, temporarily held in 88 episodes, managed with combination of reduction/hold (“mixed managed”) in 25 episodes, and unchanged in 7 episodes. Due to the variability of the platelet counts and complexity of the clinical pictures during the thrombocytopenic episodes, it was not possible to evaluate the appropriateness of a given enoxaparin dose at any specific time during the episodes. However, the mean platelet counts per cancer associated thrombocytopenia episode varied significantly across management categories (*p* < 0.001), with a median of 26,000/mcL in the group of patients for whom enoxaparin was held, compared to 41,000/mcL for those who remained on full-dose. (See Fig. 1).

The key observation was that with application of this dose-adjustment strategy there were no instances of recurrent VTE during any of the thrombocytopenic episodes, and no episode of major bleeding when the enoxaparin doses had been modified per our guidelines. Only 1 episode of major bleeding was identified, which was a trauma associated retroperitoneal hemorrhage, occurring on the third day of thrombocytopenia. At the time of that event, the platelet count was 28,000/mcL and the enoxaparin dose had not yet been reduced. Lastly, CRNMB occurred in 13 of 140 cases (9.3%), of which 6 were ecchymosis, epistaxis, or gingival bleeding, recognized complications from thrombocytopenia itself. There were 10 deaths during the thrombocytopenic episodes, believed to be cancer-related, and none were known to be related to bleeding or thrombosis. At least 20 events for any given endpoint would have been required to use modeling techniques like Cox proportional hazards regression, so multivariate analysis could not be performed.

## Discussion

The goals of this QAI program were to first, document the degree of adherence to the institutional dose-adjustment guidelines, and secondly to determine if the outcomes provided reassurance as to recurrent thrombosis and major bleeding. The patients in this analysis were treated based on our previously derived internal guidelines. The patients in this cohort received enoxaparin for treatment of VTE and exhibited an episode of thrombocytopenia lasting at least 7 days. The etiology of the thrombocytopenia was usually chemotherapy or multifactorial. Hematological malignancies were over-represented, as chemotherapy regimens used for those diseases tend to be more myelosuppressive than those administered for the treatment of solid tumors.

During the periods of thrombocytopenia, variability of the platelet counts, confounding clinical status, and chemotherapy regimens made it unrealistic to anticipate day-to-day adjustments of the enoxaparin doses. Accepting dose reduction or temporary hold as adherence to the MSKCC institutional guidelines, compliance with the guidelines was observed in 95% of the episodes of cancer-associated thrombocytopenia.

There was a significant difference in mean platelet count per episode of cancer-associated thrombocytopenia across management strategies. The mean platelet count was lowest in cases managed by temporary discontinuation of enoxaparin, compared with dose reduction, and the mean was highest in the 7 episodes where the enoxaparin was not reduced. This likely reflects rational judgment on the part of the managing physicians, as to the specific dose-adjustment strategy implemented.

This QAI program provides reassurance that the institutional guidelines maintain safety and efficacy with our dose-adjustment strategy. In none of the 140 episodes of thrombocytopenia, regardless of how the enoxaparin was managed, were there recurrent VTE events. And major bleeding occurred in none of the 136 episodes managed with enoxaparin dose modification. The one major bleeding episode may be considered the “exception that proves the rule.” One patient experienced a trauma-associated retroperitoneal hemorrhage, early in an episode of cancer associated thrombocytopenia, before the enoxaparin dose had been modified.

A prospective randomized clinical trial to validate our guidelines, or any other specific dose-modification strategy for cancer associated thrombocytopenia, would be prohibitive to conduct, due to the heterogeneity of the cancer diagnoses, chemotherapy regimens, and confounding medical conditions. In the absence of such a prospective trial, our QAI project provides guidance and reassurance for the management.

Lastly, this strategy has been validated only for enoxaparin. It is possible that a similar dose modification approach may be safe and effective for other parenteral or oral anticoagulants, but we caution against making that assumption, in the absence of specific validation.
